# The Impact of Sex and Gender on the Multidisciplinary Management of Care for Persons With Parkinson's Disease

**DOI:** 10.3389/fneur.2020.576121

**Published:** 2020-09-18

**Authors:** Irene Göttgens, Angelika D. van Halteren, Nienke M. de Vries, Marjan J. Meinders, Yoav Ben-Shlomo, Bastiaan R. Bloem, Sirwan K. L. Darweesh, Sabine Oertelt-Prigione

**Affiliations:** ^1^Department of Primary and Community Care, Radboud Institute for Health Sciences, Radboud University Medical Center, Nijmegen, Netherlands; ^2^Department of Neurology, Center of Expertise for Parkinson & Movement Disorders, Donders Institute for Brain, Cognition and Behavior, Radboud University Medical Center, Nijmegen, Netherlands; ^3^Scientific Center for Quality of Healthcare (IQ Healthcare), Radboud Institute for Health Sciences, Radboud University Medical Center, Nijmegen, Netherlands; ^4^Department of Population Health Sciences, University of Bristol, Bristol, United Kingdom

**Keywords:** Parkinson's disease, multidisciplinary care, sex factors, gender, disease progression, male, female, caregivers

## Abstract

The impact of sex and gender on disease incidence, progression, and provision of care has gained increasing attention in many areas of medicine. Biological factors–sex–and sociocultural and behavioral factors–gender–greatly impact on health and disease. While sex can modulate disease progression and response to therapy, gender can influence patient-provider communication, non-pharmacological disease management, and need for assistance. Sex and gender issues are especially relevant in chronic progressive diseases, such as Parkinson's disease (PD), because affected patients require multidisciplinary care for prolonged periods of time. In this perspective paper, we draw from evidence in the field of PD and various other areas of medicine to address how sex and gender could impact PD care provision. We highlight examples for which differences have been reported and formulate research topics and considerations on how to optimize the multidisciplinary care of persons with PD.

## Introduction

Sex and gender impact disease incidence, progression, and provision of care in different medical disciplines (1). “Sex” differences are based on biological variations due to differences in genetics, hormones, and physiology. “Gender” differences are rooted in different expressions of identity, adherence to norms, and socially defined behaviors (2). Sex can impact the biological bases of disease progression, response to diagnostics, and therapies, while gender can influence access to healthcare, coping with disease, compliance with therapies, and patient-provider communication. Taken together, these aspects warrant consideration in the provision of care to people living with a disease.

The influences of sex and gender on care delivery are especially relevant for chronic diseases that are characterized by a heterogeneous and progressive spectrum of clinical features. A prime example of such a disease is Parkinson's disease (PD), which is the second most common neurodegenerative disease worldwide and which demonstrates a rapidly rising prevalence (3). PD is partially characterized by motor features, but affected persons typically also experience a highly variable combination of non-motor features. Given the multifaceted and heterogenous nature of the disease, care delivery to people with PD typically involves healthcare professionals from a wide range of different professional disciplines to accommodate the specific clinical features, needs and coping styles of a person with PD (4–7). Ideally, any person with PD should be treated by a diverse, multidisciplinary team, consisting of a general practitioner, neurologist, PD nurse specialist, physiotherapist, occupational therapist, speech- and language therapist, neuropsychologist, dietician, or other healthcare professionals, depending on the needs of the patient (7).

At the time of clinical diagnosis, differences in the prevalence of motor and non-motor features might exist between men and women with PD. For instance, men might experience more rigidity and women more tremor (8). As the disease progresses, sex, and gender differences can emerge in the incidence of clinical features, such as postural instability or depressive symptoms (8, 9). In addition to these differences in clinical phenotype, coping styles may also vary between men and women with PD (10). Given this broad spectrum of potential differences, the consideration of sex- and gender-specific problems and needs of people with PD appears to be essential to provide personalized care. However, to date, empirical insight on the influence of sex and gender on disease progression and care for people with PD remains scarce.

This perspective paper addresses how sex and gender may impact care for people with PD, drawing from both the PD literature as well as from other fields of medicine. We will specifically focus on the following domains: (1) motor features, (2) non-motor features, (3) lifestyle, and (4) coping and informal care. To illustrate the potential impact of sex or gender, we highlight examples for which differences have been reported in PD, although the level of evidence varies substantially. For each section, the reviewed data on sex and gender differences in PD are summarized, and considerations for multidisciplinary and sex- and gender-sensitive care for people with PD are highlighted.

## Sex and Gender Aspects in PD

### Sex and Gender Aspects in Motor Features

PD is primarily known as a clinical syndrome described as “Parkinsonism,” which entails bradykinesia in combination with at least one of the following: resting tremor, rigidity, or postural instability (11, 12). As the disease progresses, people with PD are prone to develop fluctuations in motor impairments related to dopaminergic therapy, as well as to freezing of gait (13). Several differences in motor features between men and women with PD have been reported and have been summarized elsewhere (8, 14, 15). However, the relevance of these differences for care provision to people with PD remains largely unknown.

The potential impact of sex or gender differences on multidisciplinary care for mobility impairments comes from other fields of medicine, such as recent recommendations for osteoporosis screening guidelines based on underlying sex differences (16). Osteoporosis predominantly affects postmenopausal females (17) but also impacts many elderly males (18, 19). Given the higher mortality of men with bone fractures, several osteoporosis, and endocrinology societies now recommend screening in all men above 65 or 70 years (19, 20), but this recommendation is not routinely implemented in clinical practice (16).

Similarly, it is possible that sex or gender differences in the prevalence of common motor features in PD may influence clinical recommendations in the future. At the moment, however, several gaps in empirical evidence hamper development of such sex- and gender-sensitive guidelines. In Table 1, we highlight key questions that, once addressed, could guide the implementation of sex- and gender-sensitive approaches to care for people with PD.

**Table 1:** Considerations for sex- and gender sensitive multidisciplinary PD care.

**Domain**	**Feature(s)**	**Reported to be more common in**	**Possible sex- and gender sensitive care intervention(s) for this feature**	**Key questions that could guide sex- and gender-sensitive approaches**
Motor features	Poor balance	Women	• Referral to (technology-assisted) balance training interventions	• Are differences between men and women taken into account when assessing the effectiveness of balance training intervention? • Do men and women prefer different features in technology-assisted balance training interventions?
	Dyskinesia	Women	• Deep brain stimulation	• What are the underlying reasons for delayed access to deep brain stimulation surgery, on average, in women compared to men? • Do underlying gender-biases influence the shared decision-making process concerning deep brain stimulation surgery?
Non-motor features	Impulse control disorders	Men	• Reduction or discontinuation of dopaminergic therapies • Cognitive behavior therapy	• Are gender differences in ICBs due to different disease entities or socially-accepted gender behaviors? • How are patients addressed and informed about sex differences in response to dopamine replacement therapies? • Do sex or gender predict outcome in psychotherapy interventions such as cognitive behavior therapy?
	Episodes of depression and anxiety	Women	• Referral for coping skills training e.g: mindfulness-based interventions • Social support interventions	• Do screening measures for depression and anxiety take differences in gender roles into account? • Do gender traits predict or affect the responsiveness to depression and anxiety care interventions?
Lifestyle	Weight loss related impairment	Men	• Regular weight self-monitoring • Development and regular review of diet plan	• Are differences in food choices and practices between men and women taken into account in weight monitoring? • Do sex and gender aspects contribute to differences in food intake and processing?
	Limited physical activity	Women	• Exercise enhanced by motivational app elements • Physical exercise interventions	• Do exercise apps take different drivers and motivations for exercise between men and women into account? • Do exercise apps take gender-specific triggers and rewards into account in their design?
Care support	Less informal care resources	Women	• Proactive identification of social network and care capacities of the patient • Referral to social support interventions/ cognitive behavioral therapy	• Are social support interventions taking gender-specific drivers and motivators into account? • Are there gender differences in social support needs and social support perspection and how are these taking into account?
	Higher caregiver strain	Women	• Regular screening of caregiver burden • Care giver education about disease progress, symptoms and experiences	• Do screening measures of caregiver burden take gender differences in caregiver experiences into account? • Are there gender differences in information and education needs about disease progression and (advanced) care planning?

An illustration of the current gaps in knowledge is the recent observation that postural instability appears to be more common among women with PD than among men (8, 21). This observation is based on a few relatively small studies, rendering uncertainty on whether this reflects a true sex difference in the prevalence of this feature. If larger studies replicated this finding, it would encourage preferential referral of women with PD to a physiotherapist for preventive and symptomatic interventions, such as technology-assisted balance training. But for this selective referral to be effective, we also need insight on whether the effectiveness of symptomatic interventions differs between men and women with PD. Future studies should be adequately powered to examine clinically meaningful effect modification by gender, which requires larger sample sizes.

Furthermore, knowledge about the impact of gender-specific differences in activities of daily living (ADL) among people with PD is relatively scarce. The available literature, however, suggests that causal influences on ADL may differ substantially by gender. For instance, women report greater difficulty shopping and cleaning compared to men with PD, highlighting not only the practical consequences of mobility impairment, but also its gendered dimension (22). If these differences are replicated in other studies, this would encourage the development of gender-sensitive targeted occupational therapy interventions for ADL impairment (23). Taken together, empirical evidence for targeted care interventions which consider sex and gender differences in mobility impairment could eventually influence clinical guidelines for people with PD.

An additional area of potential sex- or gender-related influences on care revolves around interactions between patients and healthcare professionals. In the field of surgery, two gender-related factors affect the indication for total joint arthroplasty (24): less referral of women by their primary care physician, i.e., reflecting a potential bias on the side of the physician; and less requests by women to undergo surgery, i.e., bias on the side of the patient. A recent study suggests that women with PD are less likely to undergo Deep Brain Stimulation (DBS) surgery than men with PD (25, 26). This is of particular note given that the current literature suggests that women may experience a greater improvement in quality of life after DBS than men (9, 27). This imbalance needs to be further investigated to remove potential referral or request bias through targeted interventions on the provider or patient side (26, 28).

### Sex and Gender Aspects in Non-motor Features

Although PD is widely (and inadvertently) perceived as being primarily characterized by motor symptoms, non-motor symptoms are actually at least as common, and importantly, these can have a considerable impact on quality of life in persons with PD. In this section, we discuss two examples that highlight the potential impact of sex and gender differences on multidisciplinary care for people with PD: impulse control disorders and depressive symptoms.

Impulse control behaviors (ICBs) are associated with dopamine replacement therapy in PD. Overall, ICBs are generally more common in men compared to women with PD (29). However, the direction of these differences might differ by the specific type ICB: hypersexuality and gambling are more common in men, while compulsive buying is more common in women (30). Analogous differences have been reported for compulsive disorders in people without PD, with women presenting more contamination/cleaning symptoms or eating disorders whereas men more commonly present with sexual and aggressive symptoms (31, 32). It remains to be investigated if these differences are due to different disease entities or simply to socially-acceptable gendered behaviors (Table 1).

Depressive symptoms and anxiety are among the most common non-motor symptoms in people with PD (33, 34). Depressive symptoms and anxiety in PD are likely to be multifactorial, related to the influence of PD pathology and the indirect impact of impaired mobility and social isolation (35, 36). Sex differences in depression have been linked to differences in expression of susceptibility genes and hormonal influences as well as gender-related differences in reporting (37, 38). Although females and males with PD experience similar physical symptoms, the associated psychological burden appears to differ. Men primarily report difficulties in self-presentation, whereas women report greater psychological burden and larger impact on their intimate relationships (39, 40). This associates with a significant reduction in quality of life in women with PD (41). Also, higher anxiety levels have been reported in women with PD, especially in the early clinical phase of the disease (42–44).

However, to date, the impact of sex and gender differences in anxiety and depressive symptoms on care provision for people with PD has remained limited. Again, the field of PD is not unique in this regard. In 2008, the masculine depression scale (MDS) was developed to facilitate diagnosis of masculine depressive symptoms (45). A recent study found that men and women who endorse a masculine gender role are relatively more likely to display externalizing symptoms (e.g., anger, somatic symptoms, using substance, or sex to feel better) in response to negative life events, and less likely to report typical, internalizing depressive symptoms, as measured by, e.g., the widely used Beck Depression Inventory (e.g., depressed mood or crying) (46). Therefore, clinicians should be aware that individuals who strongly adhere to masculine gender roles, whether they be men or women, might display different signs and symptoms and may respond differently to behavioral interventions for depression and anxiety than individuals who adhere more strongly to a feminine gender role (Table 1).

### Gender Aspects in Lifestyle

Few differences in lifestyle between men and women with PD have been reported. In this section, we discuss two examples that highlight the potential impact of such differences on multidisciplinary care for people with PD: weight loss and physical activity.

Progressive weight loss is common among people with PD, likely due to a combination of physical inactivity (causing muscle loss), lower intake of solid foods due to oropharyngeal dysphagia and a catabolic state (15, 47). A decreased intake of solid foods may result in less consumption of fresh foods and vegetables, which leads to a risk of malnutrition (47). Researchers in other fields consistently reported healthier food choices among women compared to men, including increased consumption of fresh fruit and vegetables and reduced consumption of processed food and alcohol (48, 49). Encouragement by nutritionists of the consumption of healthy, solid, foods should consider these gender norms, as well as direct assessment of the abililty to prepare and consume foods due to disease-related physical limitations. Again, this is an area in which a gender-sensitive care intervention for people with PD could be informed by data from other fields. However, to our knowledge, no studies have examined the effectiveness of gender-sensitive approaches to nutrition among people with PD to date.

Once validated, gender-sensitive approaches may also help to better understand differences in body weight related impairments between men and women with PD. A useful example here comes from the field of cardiometabolic diseases, in which the observation of body fat distribution differences between women and men led to the identification of the hip-to-waist ratio as a better predictor of risk than BMI, especially for women (50). Among people with PD, weight loss generally associates with higher mortality and worse quality of life (51). While unexplained weight change is reported more commonly in women with PD (52, 53), clinically significant weight loss is reported to be associated with lower 1-year survival rates in men, compared to women with PD (54). Future studies should examine the sex-specific prognostic utility of weight loss among people with PD.

Gender considerations are also relevant in the context of physical activity. Women worldwide appear to engage less frequently in physical activity compared to men (55). Different drivers can modulate the uptake of physical activity in women and men with PD. Women appear to rely on enjoyment as the primary motivator while men describe self-efficacy as the primary driver for physical activity (56). In different regions, gender-related factors might also be at play. For example, in a qualitative study in Jordan, women with PD reported family commitment and support as important elements to initiate and maintain an exercise program. However, gender norms acted as barriers as unequal division of household tasks and childcare limited the time available for exercise (57). Different motivation strategies might be needed for women and men with PD and gender norms should be made explicit to reduce barriers to exercise (Table 1). Examples could be drawn from gender-sensitive programs to increase physical activity and promote healthy weight such as WISEWOMAN in the United States and Football Fans in Training (FFIT) in the UK (58, 59).

### Gender Aspects in Coping and Informal Care

Several differences in care management between men and women with PD have been reported. In this section, we discuss two examples that highlight the potential impact of such differences on multidisciplinary care for people with PD: coping strategies and informal care.

Gender can influence individual coping strategies and should be taken into account in systematically measuring differences in distress and coping (43). General studies on gender differences coping strategies are conflicting. Some authors report that women use more emotion-focused coping strategies while men prefer focusing on avoidant coping (60, 61). However, a study targeting coping strategies among people with PD reported the opposite, with women reporting more problem-focused coping strategies compared to males (10). Interestingly, less polarized gender roles might associate with better quality of life in women. Specifically, androgynous women with PD, expressing masculine and feminine personality traits equally, scored significantly better on quality of life than androgynous men with PD (62). Similar to the impact of gender roles on the reponse to negative life events in the context of depression, clinicians should be aware of the potential impact of gender roles on (in)effective coping strategies. Additionally, researchers should continue to explore the impact of different gender dimensions on coping strategies and health-related quality of life in people with PD.

In the context of informal care, women with PD report less social support and less informal caregiving resources compared to men (8). Women worldwide are still more frequently active caregivers than men, although this is changing in younger generations (63). Previous studies describe fewer negative outcomes and less impaired quality of life in male caregivers (64, 65). Women caregivers reported exhaustion, social constraints, and time limitations more frequently than men and women report more adverse consequences from the progression of the disease of their partners, such as feelings of manipulation, excessive demands, and lack of freedom (38). One study noted that women caregivers appeared to experience a higher incidence of depression and dysfunctional fear of progression compared to men caregivers (66), but another failed to find any gender differences in psychological, social, and health outcomes (67). Progression of disease and the potentially associated cognitive decline, which is higher in men with PD compared to women, also places a higher burden on caregivers with potential impact on their health (14, 68–71).

Proactive identification of social network and care capacities of the patient, for example by a PD nurse specialist, is needed to prevent gender disparaties in care support (Table 1). Furthermore, caregiver strain might affect female and male caregivers differently. This aspect should be actively explored, as caregivers might refrain from addressing it directly. Targeted options such as logistic support through social workers and social support through caregiver associations, should be discussed with caregivers. Psychological and educational support might be needed and should be proactively addressed with the caregiver (Table 1).

## Discussion

In this perspective paper, we highlight the potential impact of sex and gender on care for people with PD, and identify key knowledge gaps that hamper immediate implementation of sex- or gender-sensitive approaches. The intersection between biological differences and social norms and behaviors highlights the complexity of individualized care. Although knowledge in the area of sex and gender differences is increasing, the current state of evidence does not yet allow for specific recommendations for sex- and gender sensitive approaches for individual patients. In the case of PD, few studies have focused on the role of gender and the ones that did, lacked a clear definition of the concept of gender itself. Gender consists of several dimensions, such as identity, roles, and relations, and these should be clearly defined and operationalized when embarking into its investigation (72). As the previously described studies on quality of life demonstrated, gender rather then sex was predictive (62). This is in line with findings in the field of cardiology and highlights the continuous nature of the concept opposed to the simple man/woman dichotomy (73). More methodological precision in the analysis of sex and gender differences in PD will aid the transferability of the acquired knowledge into practical steps toward individualized care.

Furthermore, while the prevalence of PD has typically been higher in men than in women in clinical studies, population-based studies which include door-to-door screening and validation have demonstrated a markedly smaller gender difference in the prevalence of PD (3, 74). This discrepancy suggests that women with PD are not being referred to clinical settings as readily as men. In fact, a previous study showed that there is a considerable delay in referral of women with PD to movement disorder specialists (75). Furthermore, women are also underrepresented in clinical trials on PD and efforts to bridge this gender gap in future RCTs should be undertaken (76).

The present perspective has highlighted various areas in need of additional research. Gender-specific preferences and priorities in health care provision need to be further investigated. Which symptoms are more burdening for women and men with PD and which potential barriers exist toward optimal care provision? Are there gender-specific dimensions that contribute to long-term maintenance of quality of life? How do gender roles impact the patient's choices and can addressing them affect coping strategies? Answers to these important questions could support further refinement of multidisciplinary care programs tailored specifically to the needs of people with PD and remove potential unconscious gender-specific barriers.

## Data Availability Statement

The original contributions presented in the study are included in the article/supplementary material, further inquiries can be directed to the corresponding author/s.

## Author Contributions

SO-P and SD contributed to conception and design of the study. SO-P and IG performed the literature review and wrote the first draft of the manuscript. SO-P, IG, SD, and AH wrote sections of the manuscript. All authors contributed to manuscript revision, read, and approved the submitted version.

## Conflict of Interest

BB has received honoraria from serving on the scientific advisory board for Abbvie, Biogen, and UCB, has received fees for speaking at conferences from AbbVie, Zambon, Roche, GE Healthcare and Bial. The remaining authors declare that the research was conducted in the absence of any commercial or financial relationships that could be construed as a potential conflict of interest.
